# Glutathione S-Transferase Rescues Motor Neuronal Toxicity in Fly Model of Amyotrophic Lateral Sclerosis

**DOI:** 10.3390/antiox9070615

**Published:** 2020-07-14

**Authors:** Sun Joo Cha, Yeo Jeong Han, Hyun-Jun Choi, Hyung-Jun Kim, Kiyoung Kim

**Affiliations:** 1 Department of Integrated Biomedical Science, Soonchunhyang University, Cheonan 31151, Korea; cktjswn92@sch.ac.kr (S.J.C.); chj5913@sch.ac.kr (H.-J.C.); 2 Department of Medical Biotechnology, Soonchunhyang University, Asan 31538, Korea; hst000210@sch.ac.kr; 3 Dementia Research Group, Korea Brain Research Institute (KBRI), Daegu 41068, Korea; kijang1@kbri.re.kr

**Keywords:** amyotrophic lateral sclerosis (ALS), transactive response DNA-binding protein-43 (TDP-43), glutathione S-transferase (GST), oxidative stress, *Drosophila*

## Abstract

Transactive response DNA-binding protein-43 (TDP-43) is involved in the pathology of familial and sporadic amyotrophic lateral sclerosis (ALS). TDP-43-mediated ALS models in mice, *Drosophila melanogaster*, and zebrafish exhibit dysfunction of locomotor function, defective neuromuscular junctions, and motor neuron defects. There is currently no effective cure for ALS, and the underlying mechanisms of TDP-43 in ALS remain poorly understood. In this study, a genetic screen was performed to identify modifiers of human TDP-43 (hTDP-43) in a *Drosophila* model, and glutathione S-transferase omega 2 (GstO2) was found to be involved in hTDP-43 neurotoxicity. GstO2 overexpressed on recovered defective phenotypes resulting from hTDP-43, including defective neuromuscular junction (NMJ) boutons, degenerated motor neuronal axons, and reduced larvae and adult fly locomotive activity, without modulating the levels of hTDP-43 protein expression. GstO2 modulated neurotoxicity by regulating reactive oxygen species (ROS) produced by hTDP-43 in the *Drosophila* model of ALS. Our results demonstrated that GstO2 was a key regulator in hTDP-43-related ALS pathogenesis and indicated its potential as a therapeutic target for ALS.

## 1. Introduction

Amyotrophic lateral sclerosis (ALS) is a neurodegenerative disease characterized by a fatal and progressive loss of both the upper and lower motor neurons, resulting in muscle weakness, paralysis, and death within 2–5 years of the onset of symptoms [1]. ALS is classified into two forms: sporadic ALS and familial ALS. Sporadic ALS still lacks clear pathogenesis, while familial ALS is known to be inherited. A key component common to both familial and sporadic ALS pathogenesis is the transactive response DNA-binding protein-43 (TDP-43) [2]. The overexpression of the wild-type or mutant human TDP-43 (hTDP-43) in mice results in the degeneration of motor neurons [3]. The loss of TAR DNA-binding protein (TARDBP) or the mutation of TAR DNA-binding protein-43 homolog (TBPH), orthologous to hTDP-43 in *Drosophila melanogaster*, in zebrafish also results in motor neuron defects [4,5].

Oxidative stress plays a critical role in the pathogenesis of neurodegenerative diseases, including ALS. An excess of reactive oxygen species (ROS) or reactive nitrogen species (RNS) and an insufficient antioxidant defense mechanism are attributed to the oxidative stress conditions that give rise to many neurodegenerative diseases [6]. The biomarkers for antioxidant defense are altered in the peripheral tissues and cerebrospinal fluid of ALS patients. Similarly, the activity of superoxide dismutase (SOD), catalase, and glutathione reductase (GR) is decreased in the red blood cells and cerebrospinal fluid of patients with familial or sporadic ALS [7,8]. The reduced form of glutathione (GSH), a tripeptide, is known to non-enzymatically react with ROS for scavenger of free radical. A reduction in the GSH/oxidized glutathione (GSSG) ratio and GSH levels in erythrocytes has also been observed in ALS patients [9,10]. Therefore, the antioxidant defense mechanism against oxidative stress, which includes reactive species scavengers, is an important system in ALS. One of the antioxidants against ROS is glutathione S-transferase (GST). GSTs catalyze the conjugation of metabolites, xenobiotics, and endogenous or exogenous electrophilic compounds into their reduced glutathione form. GSTs play an important role in cellular oxidative damage, carcinogens, and drugs. Among the GST classes, several studies have found that the omega class GST (GSTO) is responsible for the detoxification of exogenous stress [11]. Under oxidative stress conditions, GSTO-1 overexpression in *Caenorhabditis elegans* has been found to increase the resistance to oxidative damage [12]. Besides, GstO1 in *Drosophila* has been shown to provide neuroprotection against H_2_O_2_-induced neurotoxicity via the mitogen-activated protein kinase (MAPK) pathway [13]. Moreover, in the *Drosophila* model of Parkinson’s disease (PD) induced by the loss of the *parkin* gene, GstO2 modulates the mitochondrial ATP synthase activity. The up-regulation of GstO2 restores the disassembly of the mitochondrial ATP synthase induced by the loss of the parkin in the PD fly model. [14]. Human GSTO1-1 upstream of NADPH oxidase-1 (NOX1), which induces the activation of ROS generators, modulates the Toll-like receptor 4 (TLR4) pathway. Lipopolysaccharide (LPS)-induced TLR4 activation in macrophages results in the generation of ROS. Thus, human GSTO1-1 is necessary for LPS-mediated redox and inflammatory responses in macrophages [15,16]. Indeed, both GSTO1-1 and GSTO2-2 have dehydroascorbate reductase (DHAR) activity that is responsible for maintaining ascorbic acid levels in humans, indicating their ability to protect against oxidative stress [17,18]. Furthermore, single-nucleotide polymorphisms (SNPs) in human GSTO1 and GSTO2 have been associated with the age at onset of Alzheimer’s disease (AD) and Parkinson’s disease (PD). GSTO locus variants may lower brain GSTO2 levels, consequently conferring AD risk in late-onset AD [19]. The GSTO1 D140 allele has been previously associated with the regulation of familial PD risk [20]. Moreover, GSTOs have been associated with loci and age at onset in Swedish ALS patients [21]. Therefore, GSTO has numerous possibilities and potential as the regulators in neurodegenerative diseases.

In this study, we aimed to investigate the neuroprotective function of GstOs in an hTDP-43-associated *Drosophila* model. In a neuronal and motor neuron Gal4-upstream activating sequence (UAS) system in *Drosophila*, the overexpression of GstO2 was found to markedly improve motor neuron function, including neuromuscular junction and axon morphology, in hTDP-43-induced *Drosophila* larvae and adult flies. Motor neuron function was recovered by overexpressing GstO2, which, in turn, resulted in an increased locomotive activity in larvae and adult flies expressing hTDP-43. Interestingly, GstO2 did not affect the protein expression level of hTDP-43 but acted as a neuroprotector in the hTDP-43-induced *Drosophila* model. Taken together, our findings suggested that GstO2 was a novel modifier of hTDP-43-related ALS pathogenesis.

## 2. Materials and Methods

### 2.1. Fly Stocks

All stock flies were kept under normal conditions with access to standard food at a normal temperature (25 °C) and normal humidity (60%). Crosses between flies were performed according to the standard procedure, and progeny was raised at a normal temperature or 29 °C. The *UAS-hTDP-43* line was gifted by Nancy M. Bonini (University of Pennsylvania, Philadelphia, PA, USA). The *UAS-GstO2* line has been described previously [14,22]. The pan-neuronal driver, *elav-Gal4*, the motor neuron-specific driver, *D42-Gal4*, and the eye-specific driver, *GMR-Gal4* lines were obtained from the Bloomington *Drosophila* Stock Center. *W^1118^* flies were used as a control.

### 2.2. External Eye Imaging

The heads of the male flies were glued to glass slides for the imaging of the external eye phenotype. The images of eye phenotype were captured using a Leica MZ10 F stereomicroscope equipped with a Leica DFC450 digital camera. Adobe Photoshop 7.0 imaging software was used to create the final images. To analyze pigment loss in a quantitative manner, a comparable area of eyes for each genotype was selected. Quantification of white regions in the selected area of eyes was analyzed using the ImageJ software.

### 2.3. Locomotive Activity

To measure the locomotive activity of the larvae, we prepared 2% grape juice-agar plates. Third instar larvae were carefully washed with PBS to remove any remnants of food medium and dried on filter paper. The larvae of each genotype were placed on a plate at room temperature and allowed to crawl for 60 s. For the quantification of larvae movement, the larvae tracking lines were drawn for the measurement of the moving distance in ImageJ software. At least, 10 larvae of each transgenic line were used to obtain results.

To perform the adult climbing assay, the male flies raised at 29 °C were collected and placed in vials (n = 20 per vial). The flies were transferred to an empty vial for 1 h at room temperature to allow for adaption to the environment. The vials were tapped gently for the flies to drop to the bottom of the vial. Then, using the fly characteristic against geotaxis, the number of flies that climbed to the top of the vial within 10 s was counted. This experiment was repeated at least 5 times independently for each genotype.

### 2.4. Immunohistochemistry

For the analysis of the larval neuromuscular junction, the third instar larvae were dissected on Sylgard plates using PBS and fixed with 4% formaldehyde in PBS for 20 min. The dissected larvae were washed in PBS containing 0.1% Triton X-100 (PBT) and blocked with 3% BSA in PBT. The samples were stained with anti-HRP-FITC (1:200; Jackson Immuno Research Laboratories, West Grove, PA, USA) overnight. After mounting the larvae on glass slides, the larval preparations were imaged using a DE/LSM710 NLO Carl Zeiss confocal microscope (Oberkochen, Germany).

To visualize the morphology of the axons, the legs of male flies were dissected in PBS and fixed with 4% formaldehyde in a fixation buffer (100 mM PIPES, 1 mM EGTA, 1% Triton X-100, and MgSO_4_, pH 6.9) for 30 min. Then, the samples were washed in a washing buffer (50 mM Tris-HCl, 150 mM NaCl, 0.1% Triton X-100, and 0.5 mg/mL BSA, pH 6.8) and mounted on glass slides. Images were acquired using a Carl Zeiss LSM710 confocal microscope (Oberkochen, Germany). The number of pixels was calculated using ImageJ software for each of the axon areas from femur regions of adult fly legs.

### 2.5. Western Blot Analysis

Protein was extracted by homogenizing the heads or legs of the flies using lithium dodecyl sulfate (LDS) sample buffer (Thermo Fisher Scientific, Waltham, MA, USA). The extracted protein was separated using a 4–12% gradient SDS-PAGE gel (Invitrogen, Carlsbad, CA, USA) and transferred to PVDF membrane (Millipore, Burlington, CA, USA). The membrane was blocked with 4% non-fat dry milk in TBS containing 0.1% Tween-20 (TBST) and incubated with primary antibodies overnight. Primary antibodies, including rabbit anti-TARDBP (1:1000; Proteintech, Chicago, IL, USA), rabbit anti-*Drosophila* GstO2 (1:1000; [14]), and rabbit anti-β-actin (1:5000; Abcam, Cambridge, UK), were used. The membranes were washed using TBST and incubated in the secondary antibody, goat anti-rabbit IgG HRP conjugate (1:2000; Millipore, Burlington, CA, USA). Protein detection was performed using an ECL-Plus kit (Thermo Fisher Scientific, Waltham, MA, USA).

### 2.6. ROS Measurement

The third instar larvae were dissected on Sylgard plates using PBS buffer. The dissected larvae were incubated with 10 μM 2′,7′-dichlorofluorescin diacetate (DCF-DA) (Sigma-Aldrich, Louis, MO, USA) for 30 min at 37 °C. The signals were observed using an MZ210F Leica fluorescence microscope (Wetzlar, Germany). Larval brains were outlined manually. The average pixel intensity of the signal in the whole brain area was measured and analyzed using the ImageJ software.

### 2.7. Analysis of Quantification and Statistics

Data collection and analysis were acted blindly. All experimental data were organized and analyzed in GraphPad Prism 8.0 using the Student *t*-test. The number of flies used, replicates, and statistical significance (*p*-values) are specified in the appropriate figure legends and methods. Significance was considered by *p*-values less than 0.05. Data were quantified and represented as the mean ± SEM.

## 3. Results

### 3.1. Overexpression of GstO2 in Eyes Recovered Retinal Degeneration Induced by hTDP-43

**Figure 1:**
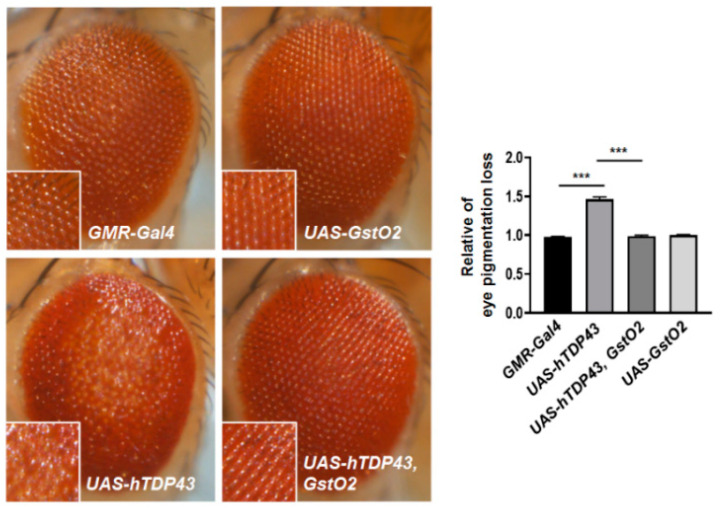
The expression of glutathione S-transferase omega 2 (GstO2) resulted in alterations of human transactive response DNA-binding protein-43 (hTDP-43)-induced retinal degeneration. Magnified images show pigment loss of each genotype. The external eye phenotypes of *GMR-Gal4* control flies and flies expressing GstO2 alone were normal. The overexpression of hTDP-43 with the *GMR-Gal4* driver induced a rough eye phenotype, including the consistent loss of eye pigmentation. The degeneration of the external eye phenotype was rescued by the co-expression of hTDP-43 with GstO2. Quantification of pigment loss in the selected area (magnified view) for each genotype was performed by ImageJ software and normalized to the value of the *GMR-Gal4* control flies. Statistical significance was determined using the unpaired Student *t*-test (*** *p* < 0.001, n = 10 for each genotypes). Error bars indicate the standard error of the mean (SEM).

Several studies have suggested that GSTO is a potential novel regulator of neurodegenerative diseases, including AD, PD, and ALS. However, the functions of GSTO in the ALS model are not yet fully understood. To investigate the relationship between GSTO expression and ALS pathogenesis, we performed a genetic interaction study using an hTDP-43-expressing fly model. Using the eye-specific Gal4 driver, glass multiple reporter (GMR)-Gal4, we assessed the genetic interactions between hTDP-43 and Drosophila GstO genes. Our results showed that GstO2, an enzymatic antioxidant, was a genetic suppressor in hTDP-43 expressing flies (Figure 1). The expression of hTDP-43 in the eyes led to retinal degeneration phenotypes, such as the loss of eye pigmentation and the disruption of the ommatidia structure. On the other hand, the co-expression of GstO2 and hTDP-43 in Drosophila eyes significantly recovered retinal disruption and increased eye pigmentation, similar to control flies. Our results suggested that GstO2 interacted genetically with hTDP-43 as a suppressor of hTDP-43-induced toxicity.

### 3.2. Defective Neuromuscular Junction (NMJ) and Motor Neurons Induced by hTDP-43 Were Recovered by GstO2 Overexpression

**Figure 2:**
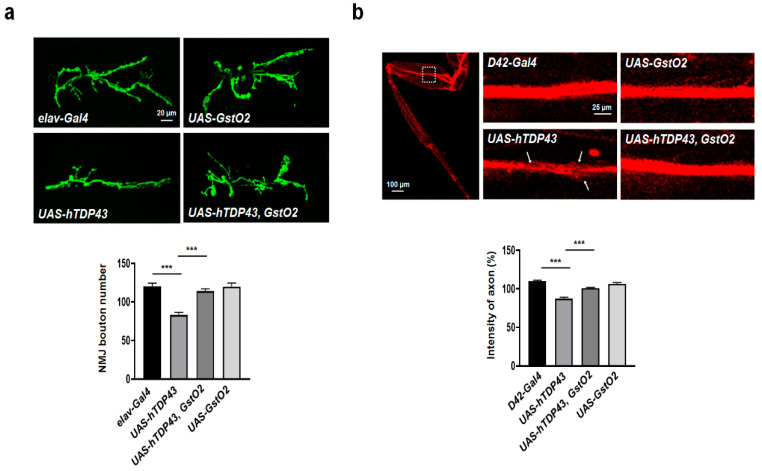
hTDP-43-induced degenerative motor neuron and loss of neuromuscular junction (NMJ) were suppressed by expressing GstO2. (**a**) Representation of NMJ boutons labeled with anti-HRP-FITC at muscle 6/7 of segment 3 and quantification of the total number of boutons. Compared with the *elav-Gal4* control larvae, hTDP-43 overexpression resulted in a significant reduction in the number of boutons. In the larvae co-overexpressing hTDP-43 and GstO2, the number of synaptic boutons was restored to a number similar to that of in control larvae. The quantification of data shows a genetic interaction between hTDP-43 and GstO2 on the total number of boutons per muscle area on muscle 6/7 of A3. Statistical significance was determined using the unpaired Student *t*-test (*** *p* < 0.001, n > 20 for each genotypes). Error bars indicate SEM. (**b**) Representative leg axon bundles labeled with membrane-tethered red fluorescent proteins (mCD8-RFP) in motor neurons specific to the *D42-Gal4* driver. The white box indicates the region used for axon images. The expression of hTDP-43 in the motor neurons of fly legs showed severe axon degeneration, denoted by white arrows. The density of mCD8 signals in the axon area of the adult fly femur (dotted box) was suppressed by GstO2 overexpression. The quantification was performed by ImageJ software and normalized to the value of the *D42-Gal4* control flies. Statistical significance was determined using the unpaired Student *t*-test (*** *p* < 0.001, n > 10 for each genotypes). Error bars indicate SEM.

To determine whether the expression of GstO2 is able to restore the neurotoxic phenotype of hTDP-43-expressing flies, we generated flies with UAS-hTDP-43, UAS-GstO2 transgenes, and pan-neuronal specific Gal4 driver, elav-Gal4. We investigated the morphology of NMJ in hTDP-43-expressing flies and measured the number of NMJ boutons by staining muscle 6/7 in segment 3 with anti-HRP-FITC. NMJ boutons facilitated the presynaptic terminal transmission of motor neurons. A similar number of the boutons was observed in control larvae, elav-Gal4, and GstO2 single overexpressing larvae (Figure 2a). Overexpression of hTDP-43 significantly reduced the total number of boutons at NMJs in hTDP-43-expressing flies compared with that in the control flies. Furthermore, co-overexpression of GstO2 with hTDP-43 suppressed the loss of NMJ boutons (Figure 2a). These findings demonstrated that GstO2 directly affected the toxicity of hTDP-43 in neurons.

Axonal degeneration in neuronal cells is the hallmark of neurodegenerative disease. To visualize the axons of motor neurons in adult Drosophila legs, we used flies expressing membrane-tethered red fluorescent proteins (mCD8-RFP) under motor neuron-specific gal4 driver, D42-Gal4. The overexpression of hTDP-43 led to severe degeneration in the axons of motor neurons in legs compared with the control, D42-Gal4 (Figure 2b; labeled by mCD8-RFP, with white arrows denoting the overexpression of hTDP-43 in motor neurons). However, co-overexpression of hTDP-43 with GstO2 suppressed the impairment of axonal degeneration in motor neurons (Figure 2b). Taken together, these findings indicated that GstO2 provided neuroprotection against the pathogenesis of TDP-43-associated proteinopathies.

### 3.3. GstO2 Restored Defective Locomotive Activity in Motor Neuron-Specific hTDP-43-Induced Flies

**Figure 3:**
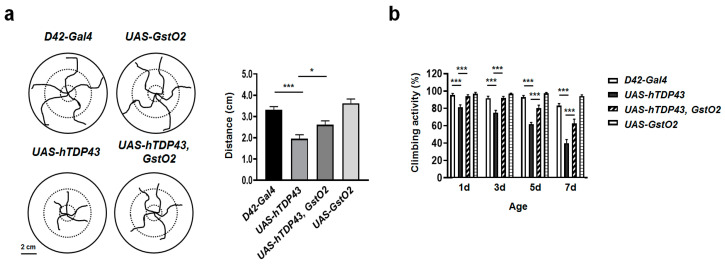
The up-regulation of GstO2 in motor neurons alleviated the motility deficits caused by hTDP-43. (**a**) Schematic illustration and quantification of larvae crawling traces from 60 s trials. The crawling distance of larvae overexpressing hTDP-43 decreased compared to the control larvae. Overexpression of GstO2 suppressed defective locomotive activity in hTDP-43-induced larvae. Overexpression of GstO2 alone had no effect on crawling activity. Statistical significance was determined using the unpaired Student *t*-test (* *p* < 0.05; *** *p* < 0.001, n > 10 for each genotypes). Error bars indicate SEM of three independent experiments. (**b**) The ratio of climbing activity in adult flies was grown at 29 °C. hTPD-43 expressed in motor neurons caused severe motility deficits in adult flies in an age-dependent manner. The overexpression of GstO2 in adult flies counteracted the reduction in climbing ability caused by hTDP-43 expression. The *D42-Gal4* control flies and flies expressing GstO2 alone showed no defects in locomotion. Statistical significance was determined using the unpaired Student *t*-test (*** *p* < 0.001, n = 20 for each genotypes). Error bars indicate SEM of more than five independent experiments.

The defective axons of motor neurons and NMJs induced by hTDP-43 expression might result in locomotor defects. GstO2 alleviated the hTDP-43-mediated degeneration of the motor neurons and NMJs, which led to the recovery of locomotor defects, as shown in Figure 2. To confirm this, we assayed locomotor ability using larva and adult flies. We generated flies expressing hTDP-43 and GstO2 in motor neurons using the motor neuron-specific gal4 driver, D42-gal4. First, we quantified the crawling activity of the larvae by placing the larvae on a grape juice-agar plate for 60 s and measuring their crawling traces. Larvae overexpressing hTDP-43 showed a reduction in crawling distance compared to the crawling distance of the control larvae. A shorter crawling distance induced by hTDP-43 was indicative of impaired locomotive activity. By contrast, the distance crawled by larvae co-expressing hTDP-43 and GstO2 was longer than the distance of larvae overexpressing hTDP-43 alone (Figure 3a).

Next, we examined the climbing activity of adult flies, as described in Section 2.3. The degenerated locomotive activity was observed in flies overexpressing hTDP-43 in an age-dependent manner. On the other hand, GstO2 overexpressed in hTDP-43-induced flies mitigated the deficits observed in the climbing ability of adult flies (Figure 3b). Our results indicated that GstO2 alleviated hTDP-43-induced motor neuronal toxicity and also suppressed locomotor dysfunction.

### 3.4. GstO2 Did Not Affect the Levels of hTDP-43 in Motor Neurons

hTDP-43 is the DNA/RNA binding protein predominantly localized in the nucleus. hTDP-43 mutations induce ALS through the mislocalization of hTDP-43 to the cytoplasm from the nucleus. hTDP-43 mutation proteinopathy has been observed in both familial and sporadic ALS patients [2,3]. Therefore, the stability of hTDP-43 is one of the critical therapeutic mechanisms of hTDP-43-induced ALS.

**Figure 4:**
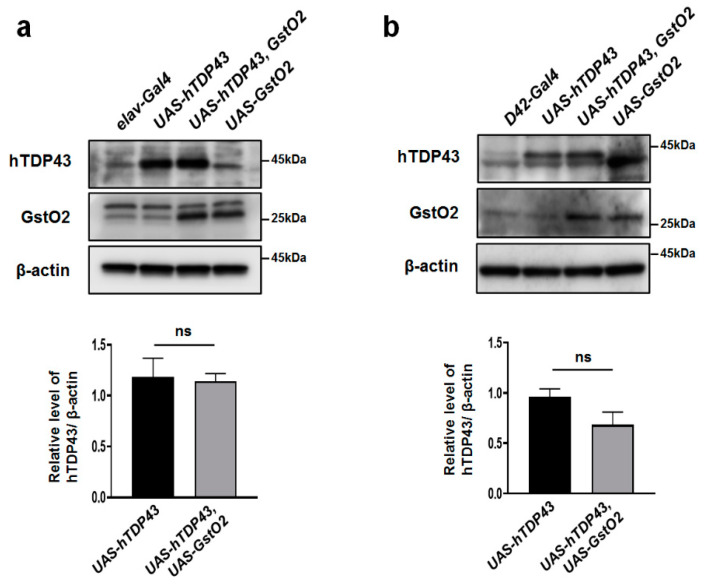
The level of hTDP-43 protein had no effect on pan or motor neurons after GstO2 overexpression. (**a**) Overexpressing GstO2 in pan neurons with *elav-Gal4* did not affect the hTDP-43 protein level. (**b**) The up-regulation of GstO2 using motor neuron-specific *D42-Gal4* driver also had no effect on the hTDP-43 protein expression level. β-actin was used as a loading control. The hTDP-43 expression level was normalized to β-actin. Statistical significance was determined using the unpaired Student *t*-test (ns, not significant). Error bars indicate SEM of three independent experiments.

We evaluated the expression level of hTDP-43 protein to determine the stability of the protein. The elav-gal4 and D42-gal4 driver were used to express hTDP-43 and GstO2, specifically in pan and motor neurons. Interestingly, the up-regulation of GstO2 in pan neurons (Figure 4a) and motor neurons (Figure 4b) did not affect the expression level of hTDP-43 protein. These results indicated that GstO2 overexpression suppressed hTDP-43 toxicity, which led to defects in motor neurons, NMJs, and locomotive activity, without reducing the level of TDP-43 protein.

### 3.5. GstO2 Reduced hTDP-43-Induced Intracellular ROS Levels in Neurons

**Figure 5:**
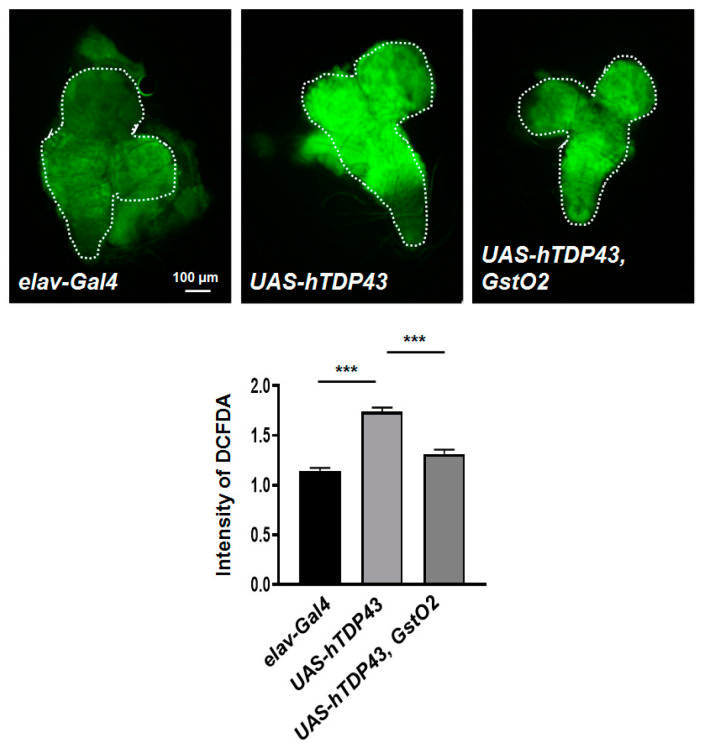
GstO2 reduced the intracellular reactive oxygen species (ROS) generation induced by hTDP-43 in neurons. 2′,7′-dichlorofluorescin (DCF) fluorescence (green) indicates the presence of ROS. ROS levels in hTDP-43-overexpressing larvae increased compared to the control larvae. Overexpression of GstO2 suppressed ROS production in hTDP-43-induced larvae. Quantification of DCF fluorescence intensity in whole brains (dotted line) was performed by ImageJ software and normalized to the value of the *elav-Gal4* control flies. Statistical significance was determined using the unpaired Student *t*-test (*** *p* < 0.001, n > 6 for each genotypes). Error bars indicate SEM.

To examine the protective mechanism of GstO2 on hTDP-43 toxicity, we measured intracellular reactive oxygen species (ROS) in the larval brain by the redox-sensitive fluorophore DCF-DA staining. ROS levels in neurons significantly increased in hTDP-43-expressing flies. Importantly, GstO2-co-overexpressing flies showed a marked decrease in intracellular ROS generation (Figure 5). These results suggested that GstO2 reduced ROS production induced by hTDP-43 in Drosophila.

## 4. Discussion

TDP-43 is a DNA/RNA binding protein encoded by a pathogenetic gene of ALS. There is evidence of the onset of ALS as a result of the presence of cytoplasmic inclusions in TDP-43 mutants in both familial and sporadic ALS patients. Moreover, some research has suggested that wild type and mutant hTDP-43 can result in a shortened lifespan, damaged synaptic morphology, degenerative motor neurons, and defective locomotion. In the present study, we confirmed that overexpressing the hTDP-43 wild type in *Drosophila* resulted in the development of degenerative motor neuronal axons. Moreover, hTDP-43 overexpression was associated with a reduced number of NMJ boutons, as well as the impairment of locomotor activity in the *Drosophila* model. These results indicated that hTDP-43 was associated with motor neuronal toxicity in *Drosophila melanogaster*.

Identification of the novel suppressor for TDP-43-induced neurotoxicity has important implications in the development of potential therapeutic targets for ALS. Using genetic modifier screening, we identified GstO2 as a novel candidate for the modulator of our hTDP-43-induced ALS fly model. Additionally, the up-regulation of GstO2 was found to directly restore neuronal toxicity, including the loss of NMJ boutons, the destruction of motor neuronal axons, and weakened locomotive ability in hTDP-43-expressing flies. Our findings supported the possibility that GstO2 plays a critical role as a suppressor in the pathogenesis of hTDP-43-induced ALS.

The mislocalization of TDP-43 protein, or the misfolding and aggregation of TDP-43 in the cytoplasm, is a hallmark of TDP-43-induced proteinopathy. As mentioned above, TDP-43 mutations have been found to accumulate in the cytosol of patients with ALS. To evaluate the stability of hTDP-43 protein, we quantified the levels of hTDP-43 protein expression. Interestingly, the level of hTDP-43 protein expression did not change with GstO2 overexpression. These results indicated that GstO2 was a motor neuroprotector that did not affect the levels of hTDP-43 protein expression.

GSTs are well-known antioxidant enzymes. They catalyze metabolites, xenobiotics, and endogenous and exogenous toxic compounds by conjugating with glutathione. Among the classes of GSTs, the omega class of GSTs also exerts detoxification effects against various stresses, including oxidative stress. For example, GSTO-1 expression is known to counteract oxidative damage in *C. elegans*. In addition, GstO1 exerts neuroprotective function against hydrogen peroxide-induced neurotoxicity via the MAPK signaling pathway in *Drosophila melanogaster*. In the fly model of PD, GstO2 has been found to modulate the activation of mitochondrial ATP synthases. Furthermore, GstO2 exerts dehydroascorbate (DHA) reductase activity and is required for the maintenance of the concentration of ascorbic acid (AsA). Human GSTO1-1 regulates the TLR4 pathway by regulating ROS generators and LPS-induced inflammatory responses in macrophages. In humans, GSTOs play a protective role against oxidative stress, including monomethylarsonic acid (MMA) reductase and DHA reductase activity. Oxidative stress plays a critical role in the pathogenesis of ALS. In the red blood cells and cerebrospinal fluid of ALS patients, the activity of antioxidant enzymes is reduced. Additionally, the levels of the reduced form of glutathione have been found to be decreased in the erythrocytes of ALS patients. Therefore, antioxidant mechanisms are an important therapeutic target against oxidative stress in ALS.

## 5. Conclusions

In our study, GstO2 was found to be a target of antioxidant defense mechanism against oxidative stress induced by hTDP-43. We demonstrated that hTDP-43 expression resulted in motor neuronal toxicity in a fly ALS model and found that GstO2 ameliorated the degenerative and defective phenotypes induced by hTDP-43 overexpression. Taken together, our results indicated that GstO2 could be used as a novel therapeutic mediator for the treatment of neurodegenerative diseases caused by hTDP-43.
